# Results of arthroscopic treatment for anteroinferior shoulder instability using a single anterior working portal using birdbeak

**DOI:** 10.1186/s13102-023-00685-5

**Published:** 2023-07-05

**Authors:** Kamil Yamak, Onur Cetin, Omer Aydemir

**Affiliations:** 1 Buca Seyfi Demirsoy Education and Research Hospital, Izmir, Turkey; 2 Medar Atasehir Hospital, Istanbul, Turkey; 3 Lapseki State Hospital, Canakkale, Turkey

**Keywords:** Bankart lesion, Shoulder recurrent dislocation, Shoulder arthroscopy, Birdbeak

## Abstract

**Background:**

The shoulder joint is the joint with the most dislocations in all joints. The arthroscopic surgery method is considered the gold standard because it creates less soft tissue damage, shorter hospitalization and surgery time, and less restriction of movement after surgery in shoulder instability. Anterior single portal technique has become popular recently. In this study, it was aimed to evaluate the results of the anterior single portal repair technique using “birdbeak”. We try to evaluate if this technique is a reliable technique and has the same or more advantages of two portal arthroscopic surgery and make the surgery easier for surgeons.

**Methods:**

In the total of 40 patients who underwent arthroscopic surgery for traumatic recurrent anterior shoulder dislocation between January 2017 and February 2020, this study included 19 patients with the surgical technique of arthroscopic isolated anterior labrum tear repair using a birdbeak from the anterior single working portal. Clinical results were evaluated with the Simple Shoulder Test (SST), Rowe Score for Instability (RWS) and Oxford Shoulder Instability Score (OSIS) tests before and after surgery. The relationship between the time to surgery after the first dislocation and clinical outcomes was also examined in the study. Kolmogorov-Smirnov and Shapiro-Wilk tests were used to control the assumption of normality. In addition, Pearson correlation and Spearman correlation analyzes were used to test the relationship between the variables.

**Results:**

The mean follow-up period of the 19 patients included in this study was 33.1 months. The mean time to surgery after the first dislocation was 18.4 months. The mean preoperative number of dislocations was 5.3. The mean number of anchors used in the repair was 2.1. No recurrent dislocations were observed after surgery. A significant difference was observed between preoperative and postoperative SST, RWS and OSIS scores (respectively, p = 0.000 < 0.001, p = 0.000 < 0.001, p = 0.000 < 0.001). There was no statistically significant relationship between the time elapsed after the first dislocation and the postoperative SST, RWS, OSIS scores (respectively, p = 0.43 > 0.05, p = 0.39 > 0.05, p = 0.31 > 0.05).

**Conclusion:**

It has been observed that the repair technique applied using the “birdbeak” from the anterior single working portal is a successful treatment, and further studies are required due to the limited literature.

## Background

Due to the wide range of motion of the shoulder joint, it is more risky in terms of dislocation than other joints [1]. Dislocation is mostly in the form of anteroinferior dislocation after trauma and results in anterior labrum tear [2]. Many open and closed surgical techniques have been described for labrum tears resulting in anterior instability. Before arthroscopic techniques developed, open labrum repair was a choice but has been almost abandoned in the last decades. Bristow-Laterjet procedure is still widely used technique for open surgeries [3]. Nowadays arthroscopic anatomical labrum repair is accepted as the gold standard as the first-line surgical treatment, due to less soft tissue damage, shorter surgical time and hospital stay, and less postoperative range of motion [4, 5].

Various arthroscopic techniques can be applied in the surgical treatment of anterior instability due to the development of imaging techniques, assistive hand instruments and various anchors used for fixation [6, 7]. The technique with successful results described in arthroscopic bankart repair is two anterior intervention portals accompanied by a posterior imaging portal [8]. The necessity of placing 2 portals close to each other in the anterior can complicate the surgery in patients with small shoulder area and narrow joint space [9]. In this case, the anterior single portal technique can be used [10]. In previous studies in the literature on labrum repairs using an anterior single portal, “lasso suture passer” was used [11]. In our study, “BirdBeak 45^0^ angled suture passer” (Arthrex, Naples, Italy) was used.

In the literature, the number of studies on the clinical outcomes of patients who underwent anterior single portal is limited [12, 13]. The 45° BirdBeak is ideal for passing sutures. The BirdBeak passer penetrates the labrum, grasps the suture, then is withdrawn to pull the suture out the cannula [13].

**Fig. 1:**
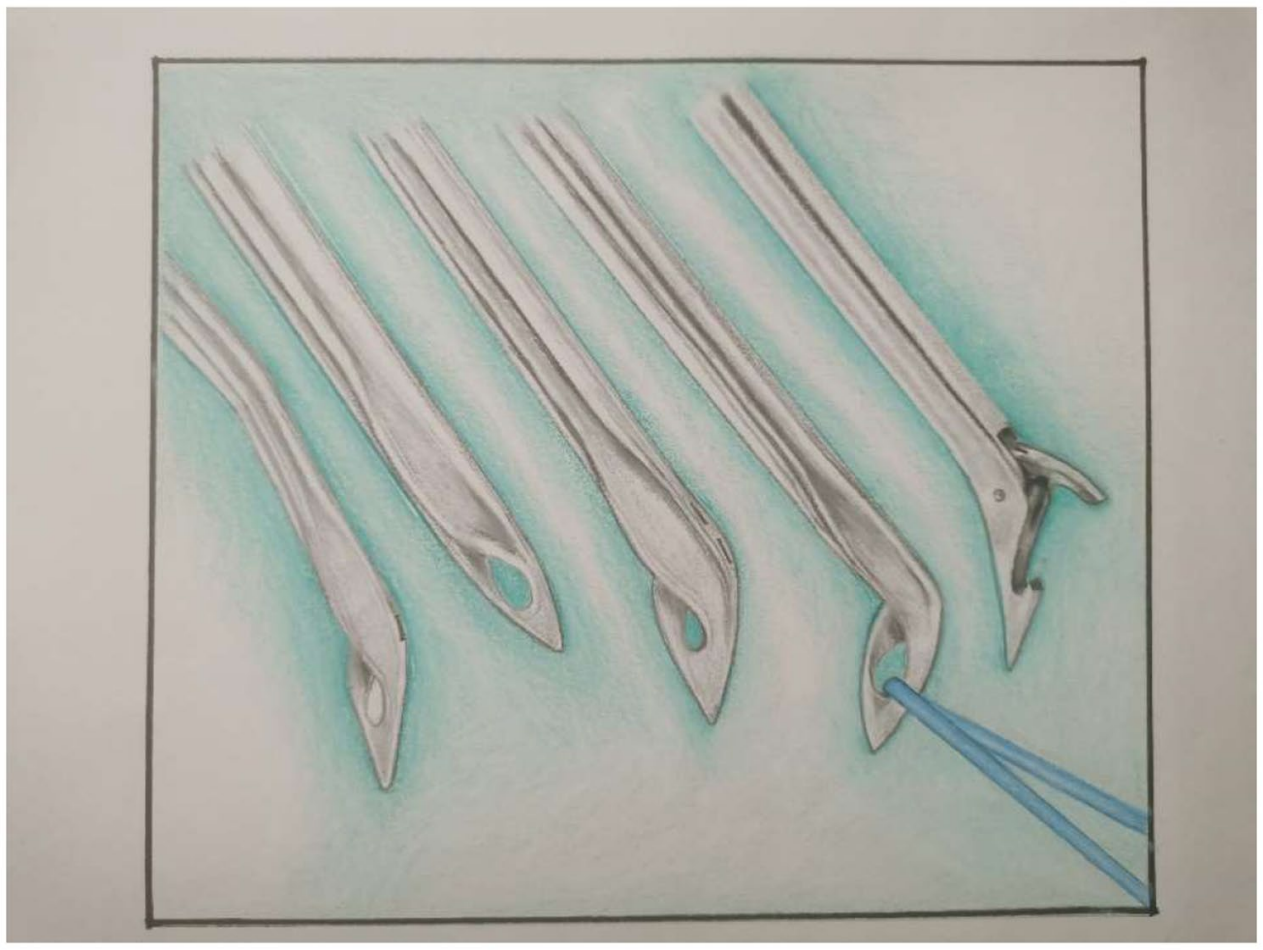
Birdbeak

The aim of our study is to evaluate the clinical results of the labrum repair performed using “birdbeak” (Fig. 1) in a single anterior portal in patients with anteroinferior instability.

## Methods

A total of 40 patients (32 men and 8 women) who underwent surgery for traumatic anterior shoulder dislocation between January 2017 and February 2020 were retrospectively scanned. After completing the post-operative physical therapy processes, 8 patients stopped coming to their follow-ups, 2 patients had Hill-Sachs lesion with glenoid bone defect, 1 patient had SLAP tear accompanying Bankart lesion. 19 patients (17 men and 2 women) who were followed up regularly, had isolated Bankart tears and were repaired using a birdbeak from an anterior single portal were included in this study.

Inclusion criteria;

- Posttraumatic bankart, perthes, ALPSA, GLAD lesions.
- Minimum 2 years of follow-up.

Exclusion criteria;

- Patients with off-track Hill-Sachs lesion.
- Glenoid defect greater than %25.
- SLAP or rotator cuff lesions accompanying Bankart lesion.
- Multidirectional and non-traumatic instability.

Initial traumatic dislocation date and the number of dislocations, apprehension test, joint laxity and neurovascular examinations were performed in all patients before surgery. Radiographs after dislocation and reduction and preoperative MRI images were evaluated.

**Fig. 2:**
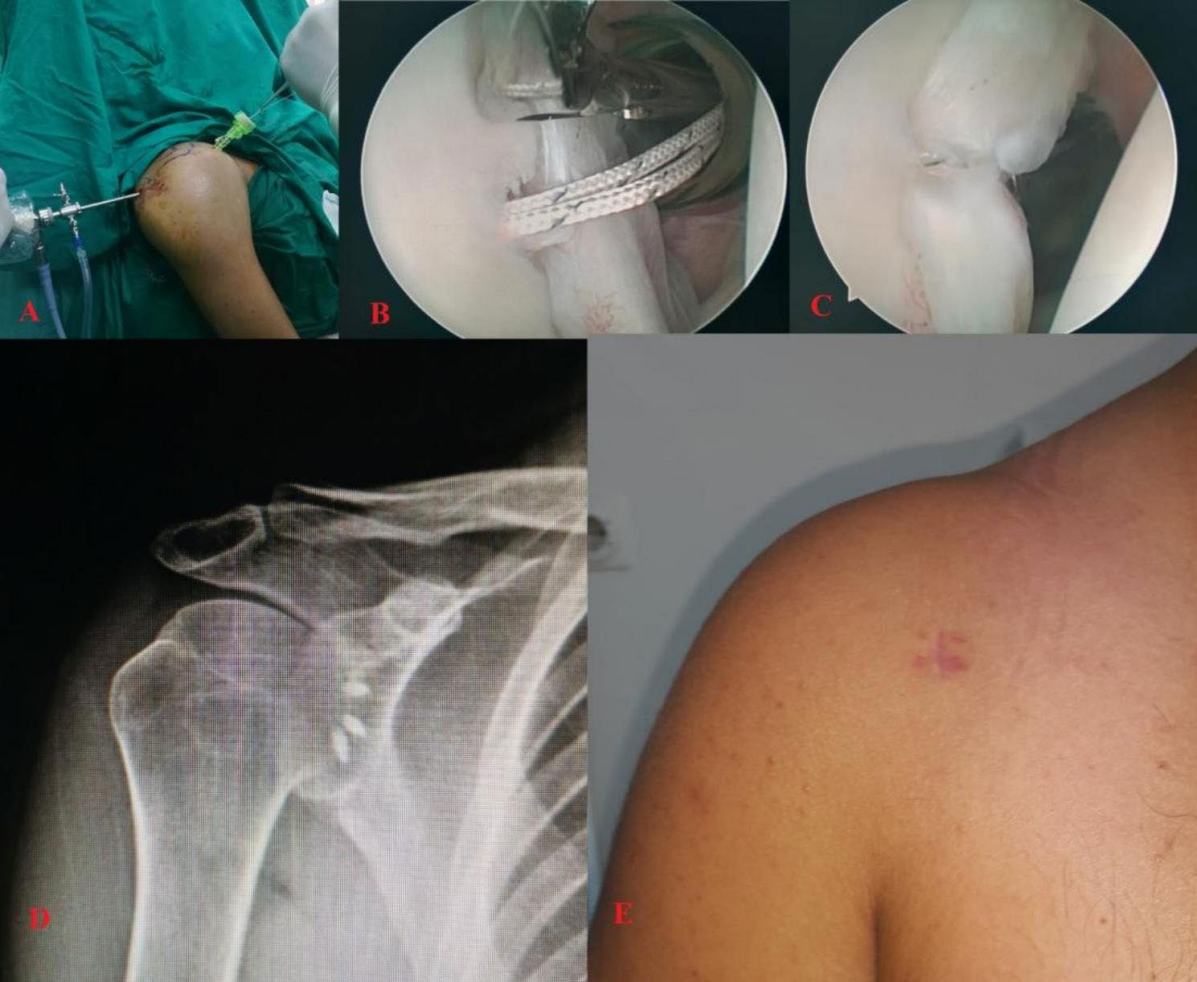
**A** Shoulder arthroscopy by opening the anterior single portal in the chaise longue position. **B** View of the birdbeak with threads through one portal after anchor placement. **C** Labrum after repair. **D** Postoperative x-ray. **E** Healed skin incision during control

The patients were operated under hypotensive general anesthesia in the beach chair position. After the posterior imaging portal was opened, the interval that would provide appropriate access from the anterior to the glenoid was evaluated. The single anterior portal opened next to the lateral coracoid, and after placing the anchor to the anterior glenoid rim, the “Arthrex 45^0^ angled BirdBeak suture passer” (Arthrex, Naples, Italy) was passed through the anterior structures and the suture was caught, and repair was performed with a sliding locking SMC suture. Care was taken not to increase the number of labral penetrations with the BirdBeak suture passer; otherwise, it was considered that it could create a large labral defect (Fig. 2).

In the first 2 weeks of postoperative period patients were followed up in velpeau bandage in internal rotation and passive pendulum movements were started in the 2nd week after the surgery. Active-assistive movements were started in the 4th week and after the 6th week patients referred to the physical therapy for strengthening. The patients were called for follow-ups at 2-week intervals until 2th month and after that, patients called back for controls at 6th month and 1st and 2nd year.

Stability and apprehension tests were evaluated in controls. Simple Shoulder Test (SST), Rowe Score for Instability (RWS) and Oxford Shoulder Instability Score (OSIS) tests were evaluated before and after surgery. SST has a purpose to assess functional disability of the shoulder and has a series of 12 questions with the answer of “yes” or “no” with 12 points of maximum [14]. OSIS assesses of pain and function of the shoulder with 12 questionaire with 5 responses each with zero to four points and 48 points maximum [14]. RWS questions shoulder stability, motion, and function. There are three sections with four answers and scores range from 0 to 100 [15].

Some descriptive statistics about the data in the study presented with mean and standard deviation values. While examining the distribution of the data, Kolmogorov-Smirnov and Shapiro-Wilk tests were used to control the assumption of normality. In addition, Pearson correlation and Spearman correlation analyzes were used to test the relationship between the variables. The p value of < 0.05 was considered significant in the study. Post-hoc power analysis of the study was calculated using G-Power version 3.1.9.7 (Düsseldorf University, Düsseldorf, Germany) and the power of our study was found to be 95%.

## Results

The mean age of the 19 patients included in the study was 25.5, and the mean follow-up period was 33.1 months. The mean time to surgery after the first dislocation was 18.4 months. The mean preoperative number of dislocations was 5.3. None of the patients underwent surgery after the first dislocation. The mean number of anchors used in the repair was 2.1. No recurrent dislocations were observed after surgery. In one patient, the scare test was positive although there was no dislocation.

**Table 1:** Statistical results of patients

	Anterior single portal (n = 19)	
	Mean ± std deviation	Preop-postop Mean ± std deviation
Age	25.58 ± 4.868	
Number of Anchors	2.16 ± 0.37	
SST score		7 ± 1.21
Preoperation	4.58 ± 1.31	
Postoperation	11.42 ± 0.61	
p	< 0.001	
RWS score		42.9 ± 8.39
Preoperation	53.42 ± 8.98	
Postoperation	96.32 ± 3.27	
p	< 0.001	
OSIS score		27.16 ± 3.1
Preoperation	19.05 ± 3.55	
Postoperation	46.21 ± 1.08	
p	< 0.001	

Preoperative and postoperative SST, RWS and OSIS test values were compared, and a significant difference was observed between preoperative and postoperative values in each test (p = 0.000 < 0.001, p = 0.000 < 0.001, p = 0.000 < 0.001, respectively). The mean postoperative SST, RWS, and OSIS test values (11.42, 96.32, 46.21, respectively) were found to be better than the mean preoperative values (4.58, 53.42, 19.05, respectively) (Table 1).

It was evaluated whether there was a significant relationship between the time elapsed after the first dislocation and the postoperative SST, RWS and OSIS scores, and no significant relationship was found between the time of dislocation and clinical scores (p = 0.43 > 0.05, p = 0.39 > 0.05, p = 0.31 > 0.05, respectively).

## Discussion

Recurrent shoulder dislocations are an important health problem that affects the young adult population and creates serious limitations in their life. Anterior instability is a broad spectrum ranging from a labral lesion involving purely soft tissue injury to instability with bone loss. The separation of the anteroinferior labroligamentous complex from the glenoid together with the periosteum, which often accompanies traumatic first dislocations, is called the classical “Bankart lesion”. Before surgery, the reason behind the instability should be clearly revealed. The osseous integrity of the glenoid, the labral-ligamentous complex that provides the centralization of the humeral head, the muscle strength that presses the humeral head to the glenoid, the joint surface and its integrity should be evaluated and the problem should be determined correctly, and the correct treatment and prevention of the problems after the treatment should be ensured.

Nowadays, minimally invasive techniques are becoming widespread in many areas of orthopedics. Shoulder arthroscopy is a treatment proven to be as successful as open surgery and has many advantages over open surgery, such as early movement, less pain, early discharge, less scar tissue [11, 16, 17]. The learning curve for arthroscopic treatments of the shoulder is long. The main problems experienced in the anterior double portal technique are the close placement of the cannulas, the loss of time to open the second cannula, the tangling and knotting of the sutures during suture transport. Compared to the anterior double portal technique, the anterior single portal technique, which is thought to facilitate the surgical technique, has recently been preferred by many shoulder surgeons. In our study, we aimed to show that in arthroscopic anterior stabilization, which is as successful as open surgery, anterior single portal repair technique using “birdbeak” is as successful and reliable as the classical anterior double portal technique.

There are a limited number of studies in the literature comparing anterior single portal and anterior double portal techniques. In recent studies, tests such as Constant Shoulder Score, RWS and OSIS were evaluated in the anterior single portal group, and no statistical difference was found in the postoperative test scores between the two groups [11, 18, 19].

In our study, we evaluated preoperative and postoperative SST, RWS and OSIS test scores. When the test results were evaluated, the results of the anterior single portal technique were found to be similar to the anterior double portal technique in the literature. From this point of view, the anterior single portal technique was thought to be as reliable as the anterior double portal technique.

Re-dislocation is the most undesirable complication after surgical treatment in patients with anterior shoulder instability. Especially in patients with multiple dislocations, bone defects reduce the success of the surgery [20, 21]. As a result of the 3-year study by Boileau et al., a 15% risk of re-dislocation was observed after arthroscopic repair, another study conducted by Gartsman et al., the risk of re-dislocation was observed as 8% [22]. In other studies, it was observed that the risk of instability increased as a result of weakening of the rotator interval [23, 24]. It can be thought that the injury in the rotator interval with the anterior double portal technique increases the risk of dislocation since it is less injured in the single portal. In our study, no re-dislocation was observed although apprehension test was positive in 1 patient who had no additional problems observed in the follow-ups.

The most technically challenging part of arthroscopic bankart repair with the anterior single portal is to properly and perfectly position the anterior portal to reach the lowest part of the torn labrum [25]. In some studies, the use of transscapular portals has been suggested to have a better view for the lower part of the lesion during arthroscopic anterior stabilization, but risks such as proximity to the axillary artery and nerve, musculocutaneous nerve and cephalic vein should be in mind [26, 27]. Failure to place anchors in a good position can result in failure of repair, recurrence of instability, and high risk of recurrent dislocations [28]. Therefore, in our study, the anterior single portal was placed just above the subscapularis muscle. With this portal placement, the lowest anchor can be placed as close to the 5 o’clock position as possible and stabilization of the glenoid labrum can be achieved [25].

Surgical intervention after the first dislocation still remains a matter of debate. In a single-blind study, a comparison was made between the patient group who underwent three weeks of immobilization and then rehabilitation treatment in case of acute first dislocation, and the patient groups who underwent arthroscopic anterior stabilization within the first four weeks; It was stated that the shoulder stability of the patients who underwent arthroscopic intervention in the first four weeks was better than the patients followed by immobilization, but the shoulder range of motion was similar in both groups [29]. None of the patients in our study underwent surgical intervention within a month after the first dislocation.

The use of “Birdbeak” has been described in arthroscopic procedures for repair of labral tears, rotator cuff tears, spinoglenoid notch cyst and SLAP [30, 31]. The “BirdBeak 45^o^ angled suture threader” (Arthrex, Naples, Italy) has an extremely sharp tip to easily penetrate soft tissue, a rigid structure that resists bending during tissue shifting, and a handle that allows easy operation from any hand position. Similar to other suture-permeable and carrier techniques, care should be taken to ensure that the number of penetrations is low and that no labral defect is created in labral repair with the “Birdbeak suture threader”. The use of a “suture lasso” (Arthrex, Naples, FL) has been described previously to capture anchor threads and pass threads through the labrum, following placement of anchors for labrum repair from the anterior single portal [25]. The use of knotless anchors has also been reported in the literature due to its advantages such as ease of use and no knots in the joint [10]. In our study, “Birdbeak” was used for anterior stabilization and labrum repair. A similar study using “Birdbeak” has not been observed in the literature. The limitations in our study are the size of the groups which was relatively small, but the statistical power was sufficient. Randomised and further follow-up studies would be needed to confirm the results of the present study.

## Conclusion

In conclusion, anterior single portal method in arthroscopic bankart repair is as effective as the classically applied anterior double portal method and its results are satisfactory. In addition, problems in the arthroscopic double portal bankart repair such as opening the second portal, carrying sutures between the portals, tangling the sutures and eventually prolonging the surgery can be eliminated and that makes the surgery easier for the surgeons.

Due to the limited publication on this subject and its relatively recent application, the literature needs to be supported with more patients and long-term follow-ups.

## Data Availability

Figures in this study can be accessed through correspondence author (Dr Onur Cetin, drocetin@gmail.com).
